# Radar Sensor Data Fitting for Accurate Linear Sprint Modelling

**DOI:** 10.3390/s24237632

**Published:** 2024-11-29

**Authors:** Daniel Geneau, Patrick Cormier, Ming-Chang Tsai, Dana Agar-Newman, Seth Lenetsky, Marc Klimstra

**Affiliations:** 1 Canadian Sport Institute Pacific, Victoria, BC V9E 2C5, Canada; 2 School of Exercise Science, Physical and Health Education, University of Victoria, Victoria, BC V8P 5C2, Canada; 3 Sport Performance Research Institute New Zealand, Auckland University of Technology, Auckland 1010, New Zealand

**Keywords:** velocity modelling, biomechanics, overground running, force velocity, power

## Abstract

Background: Accurate linear sprint modelling is essential for evaluating athletes’ performance, particularly in terms of force, power, and velocity capabilities. Radar sensors have emerged as a critical tool in capturing precise velocity data, which is fundamental for generating reliable force-velocity (FV) profiles. This study focuses on the fitting of radar sensor data to various sprint modelling techniques to enhance the accuracy of these profiles. Forty-seven university-level athletes (M = 23, F = 24; 1.75 ± 0.1 m; 79.55 ± 12.64 kg) participated in two 40 m sprint trials, with radar sensors collecting detailed velocity measurements. This study evaluated five different modelling approaches, including three established methods, a third-degree polynomial, and a sigmoid function, assessing their goodness-of-fit through the root mean square error (RMSE) and coefficient of determination (r^2^). Additionally, FV metrics (*P_max_*, *F*_0_, *V*_0_, *FV_slope_*, and *DRF*) were calculated and compared using ANOVA. Results: Significant differences (*p* < 0.001) were identified across the models in terms of goodness-of-fit and most FV metrics, with the sigmoid and polynomial functions demonstrating superior fit to the radar-collected velocity data. Conclusions: The results suggest that radar sensors, combined with appropriate modelling techniques, can significantly improve the accuracy of sprint performance analysis, offering valuable insights for both researchers and coaches. Care should be taken when comparing results across studies employing different modelling approaches, as variations in model fitting can impact the derived metrics.

## 1. Introduction

Sprint running is a crucial human locomotive task in individual- and team-sports and is therefore a critical area of interest for sports scientists [1,2,3,4,5,6]. Accordingly, maximal linear sprint profiling is a common approach to evaluate athlete sprint mechanical characteristics such as horizontal force and power, which are associated to a variety of sport-specific tasks [1,3,7,8]. A common approach to model center of mass (CoM) acceleration is through horizontal force-velocity (FV) profiling, which continues to increase in popularity due to its scope of application, ranging from athlete monitoring to rehabilitation and training program design [2,7,9]. The standard FV approach utilizes a mono-exponential velocity–time fitted model for linear sprint trials [1]. The theoretical basis of this work comes from Furusawa, Hill, and Parkinson (1927) [4], and was confirmed by Best and Partridge (1928) [10]. It was demonstrated, from first principles, to explain and predict the athlete’s ability to accelerate and achieve maximal velocity during a maximal linear sprint. While this model has been shown to be a reliable means of determining FV metrics, several mechanical and methodological assumptions exist that may challenge the appropriate use of this equation and its constituent components for all sprint modelling applications [1,11].

For example, the mono-exponential function assumes that an athlete’s largest horizontal acceleration occurs at the onset of the sprint (i.e., time = 0) followed by a consistent exponential decay [1,12]. This assumption does not fully consider potential differences between phases of the sprint that could modify athlete acceleration [6,13]. Ettema et al. (2016), in their analysis of sprint running, observed the existence of breakpoints in certain variables during the sprint acceleration phase. While their observations anecdotally suggested a “perfect” exponential increase in speed throughout the sprint, the existence of breakpoints was highlighted and considered to have been a result of sprint performance error related to training level [6], starting position variability [14], or athlete asymmetries [13,14]. The existence of these movement strategy differences during the acceleration phase observed in some athletes could support differences in a participant’s ability to produce horizontal force through their CoM [13] and may also suggest that sprint velocity data may not perfectly align with the theoretical mono-exponential function. Additionally, mono-exponential models include an asymptote on the *y*-axis indicating that the final velocity must be the maximum velocity of the trial, neglecting the possibility of athlete deceleration. This carries implications for collection protocols as well as for the post-processing methodology.

While there is value to the simplicity of the mono-exponential function to provide biomechanically relevant data, other models may provide a better fit to the velocity data as well as address some of the limitations of the standard approach. For example, Volkov et al. (1979) [5] proposed a model (bi-exponential) that allows for a velocity decay following achievement of peak velocity, a method which has been replicated in more recent work [9,15]. From a physiological perspective, maximum effort can generally only be maintained for 5–6 s [5] at which point a negative acceleration would be observed. However, depending on sprint distance and the athlete’s skill level, not all sprint trials will include velocity decay, and this model’s parameters may therefore be unnecessary. Further, this bi-exponential model has the same assumption as the mono-exponential functions, where the maximum acceleration takes place at the initial instance of the sprint followed by immediate decay, possibly limiting its applicability to certain athlete populations. Another approach is to include a time correction, or temporal offset, for the mono-exponential function when the velocity data do not begin from zero. For example, recent work by Morin et al. (2019) [12] included an estimated time-delay constant to the velocity model equation, a simple change to the standard mono-exponential model, which displayed improvements in the modelled performance [12].

Other fitting techniques, such as a polynomial model, have also been used to model object trajectory [16] and athlete sprint velocity [17,18], which eliminate many of the limitations present in exponential functions. Previous work has explored the use of a second-degree polynomial model for velocity fitting [18]; however, the numerical derivative of this function is a linear model that does not adequately represent acceleration during a sprint. As a result, the third-degree polynomial provides the appropriate degrees of freedom to appropriately model velocity and the subsequent acceleration phenomenon required to complete an FV profile. Yet, it is important to note, this model has no physiological basis, and the calculated variables do not relate to any specific physical phenomenon present during the sprint, limiting its applicability for practitioners. Other models, such as the sigmoid model, have similar flexibility to polynomial functions but have proven to be relevant for modelling biological and mechanical processes such as muscle activation profiles and wheelchair-rolling friction modelling [19,20], potentially making it more relevant to sprint performance. Further, the sigmoid model can exactly mimic the exponential relationship observed in the mono- and bi-exponential models while still allowing (but not requiring) the maximal acceleration of the trial to take place after the sprint onset.

Based on the present limitations of the mono-exponential model and the potential benefits of other FV modelling approaches, it is advisable to compare the current standard mono-exponential model to other models for the development of FV variables from sprint velocity data. Therefore, the purpose of this investigation is to compare the mono-exponential, time-corrected mono-exponential, bi-exponential, polynomial, and sigmoid methods to determine which model has the best goodness-of-fit to experimentally derived sprint velocity data. Additionally, FV metrics derived from each model will be compared. Further, to assess the degree to which each model can determine differences between fast and slow athletes, each model will be fit to the fastest and slowest athlete trials across the collection cohort and then FV metrics will be compared. The reliability of each model will also be evaluated by increasing velocity data from individual trials by percentage steps ranging from 1 to 10% change.

We hypothesize that there will be discrepancies in the goodness-of-fit to raw velocity data between different modelling techniques. Furthermore, due to these differences, we anticipate variations in the FV output measures and overall sensitivity between the velocity models.

## 2. Materials and Methods

### 2.1. Subjects

Forty-seven participants (male = 23, female = 24; age = 21.26 ± 2.23 years; height = 1.75 ± 0.1 m; mass = 79.55 ± 12.64 kg) from university ice hockey and rugby programs participated in this study. Participants ranged in their sprint ability, previous sprint training, and sporting background. All participants were free of any injury at the time of testing. Ethical approval was obtained from the University of Victoria’s Human Research Ethics Board. This study complied with the principles outlined in the Declaration of Helsinki.

### 2.2. Procedures

#### 2.2.1. Data Collection

The protocol was performed on an outdoor rubberized track surface with participants wearing standard running shoes. Each participant completed a standardized 20-minute warm-up consisting of general and dynamic movements followed by three progressive sprints at increasing intensity (60%, 70%, and 90% maximum effort) prior to their first trial. Following the warm-up, participants completed two 40-meter sprint trials with a minimum rest period of 5 min to ensure the maximal effort needed to generate an FV profile across both trials. Participants were instructed to “stand still” in a staggered 2-point stance for 2 s before commencing the sprint to avoid backwards or forwards movement (i.e., “rocking”) before the sprint and to minimize movement prior to the inflection point of the velocity (i.e., CoM displacement). For each trial, a radar gun (Stalker ATS II, Richardson, TX, USA) was set at a height of 1 m, positioned 5 m behind the participant, pointed directly at the subject’s lower back. Stalker Stats II software (Version 5.0.3.0, Applied Concepts, Dallas, TX, USA) was used to collect instantaneous velocity data for each sprint (46.875 Hz). Data were filtered before export by the software (fourth order, zero lag, Butterworth filter) in line with previous research [21]. Barometric pressure (Torr), wind velocity (km/h) relative to the sprint direction, and ambient air temperature were collected (760 Torr, 7 °C, 2 km/h blowing south to north, respectively) from the University of Victoria weather station (latitude 48.46, longitude −123.6, elevation 60 m; Vantage Pro2, Davis Instruments Corporation, Hayward, CA, USA). All sprints were run from north to south.

#### 2.2.2. Data Processing

**Figure 1:**
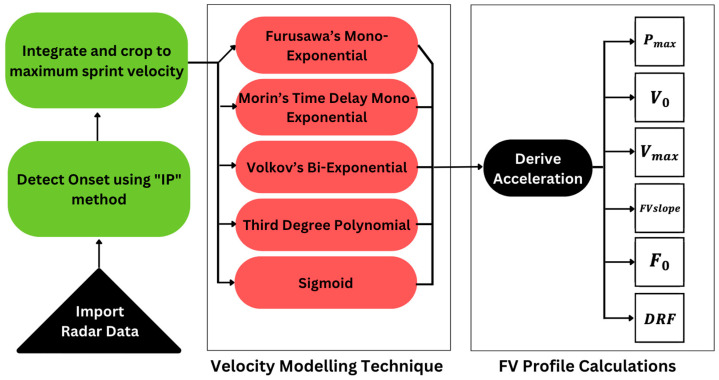
Data processing progression used to determine FV measures for each velocity-modelling technique.

All processing was performed using custom software (Python 3.2, Beaverton, OR, USA). Instantaneous velocity data were cut using detected onset values representing the initiation of the sprint and offset at the participant’s maximum velocity. Sprint onset was detected using a technique similar to the “IP” method described by Zhou and Zang (2014) [22]. This technique identifies the onset as the moment of maximum inflection from rest where the largest change from rest takes place. This was conducted by identifying the first point above 50% of the maximum velocity within a trial and creating a linear regression from that point to an instance recorded during quiet standing. This regression was then subtracted from the signal in this range, and the onset was identified as the resultant peak. All trials were then time normalized over 1000 samples to easily draw comparisons between sprint trials. Velocity models were then fit to each sprint trial and used to calculate subsequent FV measures, as outlined in Figure 1.

#### 2.2.3. Sprint Modelling

Sprint velocities were modelled with five different techniques (as outlined in Equations (1)–(5)). Two of the five models are mono-exponential curves that have been previously used within the literature and have been validated for the determination of FV measurements. The bi-exponential and third-degree polynomial fitting techniques have been used previously for velocity modelling; however, these models have yet to be applied for FV profiling. Finally, the sigmoid model has not been used for sprint modelling in any capacity. For each technique, the velocity and subsequent acceleration models were created using a custom Python script, and the parameters were estimated using nonlinear least squares regression, which were then used to derive the model’s horizontal force, velocity, power, and the ratio of forces throughout each sprint. From this, a total of six common six FV outputs were calculated—the theoretical maximum velocity at zero force $[V_{0}],$ maximum modelled peak velocity [$V_{max}$], maximum power [$P_{max}$], maximum theoretical force at zero velocity (relative to body mass) [$F_{0}$], the decrease in the ratio of force [DRF], and the force-velocity slope [${FV}_{slope}$]—to estimate each model’s parameters while accounting for horizontal aerodynamic drag force [$F_{areo}$]. Horizontal force and all subsequent FV output measures were estimated using the same methods as outlined by Samozino et al. [1], with the $F_{areo}$ being subtracted from the product of athlete mass (m) and center of mass acceleration (*a_com_*) throughout the sprint as outlined in Equations (1) and (2). In the calculation of $F_{areo}$, *k* represents the runner’s aerodynamic drag coefficient (determined using barometric pressure, temperature, and athlete stature) and *V_W_* represents the wind speed relative to the direction of sprint displacement.

(1) $$F_{areo}\left(t\right)=k\;\cdot (V_{H}\left(t\right)-V_{W})$$

(2) $$F_{H}={(a}_{com}(t)\cdot \;m)-F_{areo}(t)$$

As such, the following six FV outputs were calculated: theoretical maximum velocity at zero force [$V_{0}],$ maximum modelled peak velocity [$V_{max}$], maximum power [$P_{max}$], maximum theoretical force at zero velocity (relative to body mass) [$F_{0}$], the decrease in the ratio of force [DRF], and the force-velocity slope [FVslope]. Finally, goodness-of fit outcomes were extracted from each modelled sprint and FV measures across all trials.

(3) $$V_{H}\left(t\right)=V_{max}\left(1-e^{-t/\tau }\right)\;\mathrm{Mono-exponential}$$

(4) $$V_{H}\left(t\right)=V_{max}\left(1-e^{-(t+t_{0})/\tau }\right)\;\mathrm{Mono-exponential}\;(\mathrm{time delay})$$

(5) $$V_{H}\left(t\right)=V_{max}\left(e^{-k_{2}t}-e^{-k_{1}t}\right)\;\mathrm{Bi-exponential}$$

(6) $$V_{H}\left(t\right)=at^{3}+bt^{2}+ct+d\;\;\mathrm{Third degree polynomial}$$

(7) $$V_{H}\left(t\right)=\frac{L}{1+e^{-k(t-t_{0})}}+b\;\mathrm{Sigmoid}$$

To better assess each model’s ability to determine between-group differences, the top and bottom 25% of sprint trials (based on the 40 m sprint time) within our sample were separated into two groups. These groups were then compared to see if each velocity model adequately detects the differences between athlete performance groups. Further, the individual sprint datum was systematically modified and compared to the baseline to evaluate each model’s robustness and within-subject reliability. Trials were altered by percentage increments, ranging from 1 to 10% (i.e., the trial velocity data set was multiplied by a multiple of 1.01 to an effect of 1% change). At each stage, FV outputs were compared to the baseline measures generated from the unmodified data. The percentage at which significant change occurred for each model was reported for each respective FV variable.

**Figure 2:**
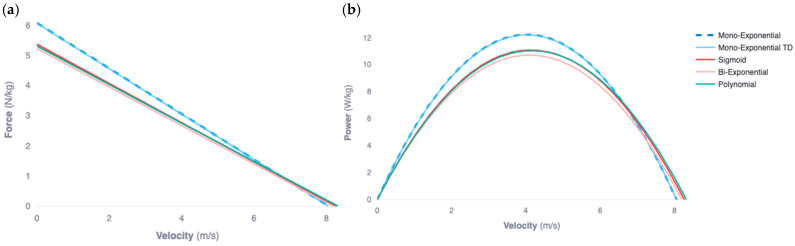
Average outcomes for both the force-velocity (**a**) and power-velocity (**b**) measures derived from each modelling technique.

**Figure 3:**
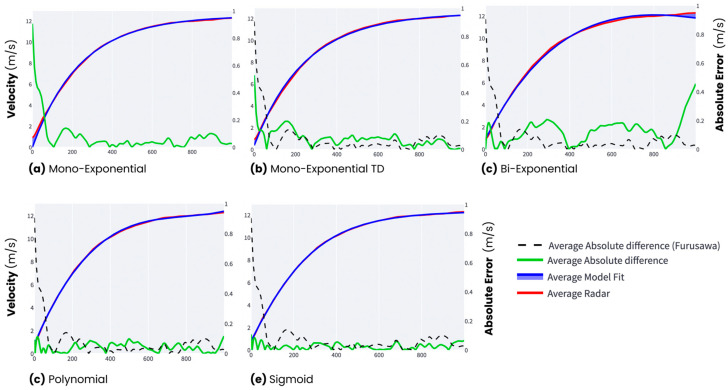
Velocity-fitting traces for each modelling technique: (**a**) mono-exponential; (**b**) mono-exponential TD; (**c**) bi-exponential; (**d**) polynomial; and (**e**) sigmoid. Average fits and radar signals are represented with blue and red lines, respectively. Similarly, model absolute error is displayed using the green line. Furusawa’s mono-exponential model error is represented in each plot using a dashed black line.

Finally, to best visualize trends across models, average radar signals were superimposed with each model’s average velocity fit. Average force velocity profiles for each modelling technique were visualized (Figure 2). Further, absolute error radar and model averages was also graphically represented (Figure 3), indicating regions of common or systematic error unique to each modelling technique.

### 2.3. Statistical Analysis

Sprint trials were evaluated for goodness-of-fit using the root mean square error (RMSE) and coefficient of determination (*r*^2^) across modelling techniques to quantify which model achieves the best velocity trace for a linear sprint relative to the radar measurement. Prior to statistical comparisons, the coefficient of determination was corrected using Fisher’s transformation to create a linear relationship between metrics. Univariate ANOVA analysis was used to compare FV metrics and goodness-of-fit parameters between models. Dunnett’s post hoc procedure was used to control for multiple comparisons, to determine significant differences between the metrics and parameters from the models against Furusawa’s mono-exponential model as reference. For assessing model between-group reliability, comparisons between the top and bottom 25% of the sprint trial groups were evaluated using independent t-test analysis. For the percentile differences, to evaluate within-cohort reliability, repeated measures ANOVA and Tukey’s post hoc analysis were used to identify if and at what percentage stage the significant changes occur in FV measures for each model.

## 3. Results

Significant differences between models were observed for both goodness-of-fit measures and all FV outputs other than $P_{max}$, $V_{0}$, and $V_{max}$. Tukey’s post hoc analysis displayed significant differences between every model, except between Furusawa’s and Morin’s models and the sigmoid and polynomial fit models. These differences can be clearly visualized in Figure 2.

Volkov’s bi-exponential model was significantly different than the mono-exponential model for all FV outputs but there were no significant differences in goodness-of-fit measures between these two models (Table 1). Similarly, Volkov’s and Morin’s time-delay models displayed highly significant differences for all FV outputs but no observable differences in the *r*^2^ or RMSE metrics. Finally, Volkov’s model showed significant differences from the polynomial and sigmoid models for goodness-of-fit measures.

Significant differences between the top and bottom 25% of sprint trials were observed for all FV outputs except DRF across all models. Further, significant differences were observed at various percentages of change across FV levels consistently across all velocity models, as seen in Table 2.

## 4. Discussion

This study compared horizontal FV modelling techniques in a heterogeneous athlete cohort and found important differences that require consideration. First, sigmoid and polynomial functions presented better goodness-of-fit to the velocity data than other approaches, supporting the use of these models alongside currently accepted techniques for horizontal FV modelling. This finding brings into question the use of current modelling approaches across differing cohorts and sprint conditions. Second, there were statistical differences between the FV metrics developed using different methods. Differences in metrics between approaches require further investigation as it is important to confirm which metrics accurately represent the force and velocity capability of the athlete associated with their maximal sprint performance. Differences in $F_{0}$ could be related to an inability of specific modelling techniques to appropriately fit different phases of the sprint. This indicates that care should be taken when comparing results from studies using different modelling approaches. Thirdly, all approaches were able to determine differences between fast and slow athlete cohorts and showed comparable reliability with small changes in data magnitude; however, the physiological and mechanical interpretation of variables present within each model vary. Overall, this study highlights important considerations for the use and selection of velocity-modelling approaches for the development of force-velocity profiles.

It is commonly accepted that the speed profile of an athlete sprinting fits an exponential function. This was first presented by Furusawa, Hill, and Parkinson (1927), and was confirmed by Best and Partridge (1928) as a basic mono-exponential function was demonstrated to predict the speed profile of an athlete performing a maximal-effort linear sprint. While the equation presented provided an important first-principles approach to sprint running dynamics and was shown to fit the experimental data, there has been no attempt to determine if it was indeed the best fit to sprint running under multiple circumstances. Only recently have limitations in the fit of this function to sprint data been addressed through parameter modifications to support its use [12,23]. For example, Vescovi et al. (2021) added a time-delay component to improve the fitting of this function to timing gate data compared to standard mono-exponential fitting. Further, there are many sprint running protocol differences that may alter the ability of an athlete to accelerate their CoM, which may also change the efficacy of a basic mono-exponential function to appropriately fit the data. Specifically, in all exponential functions, maximal acceleration is assumed to be at the onset of the sprint, and only instantaneously maintained [1,4,11,12]. However, different starting protocols (i.e., standing/block/rapid initiation) and an athlete’s sprint ability may result in different acceleration characteristics and, in turn, changes in the various phases or breakpoints of a sprint [14,24,25]. In addition, considerations such as field sport athlete’s position [26], previous sprint training, or asymmetries [13,14] may also significantly alter the acceleration and velocity profiles of an athlete during a sprint. In this investigation, there are clear areas where there is substantial error in comparing the modelled fit to the data, as seen in Figure 3. This is observed with more error at the beginning of the sprint with the standard mono-exponential and mono-exponential time-delay functions, while the polynomial and sigmoid fits performed with less error in this sprint region. This could be due to the specific protocol used in this experiment or the technical ability of the athlete cohort used. While the mono-exponential function may be an ideal fit to a theoretically optimal acceleration profile during a sprint, it is important to ensure that the model used is the best fit to the experimental data, which, in this cohort, was both the polynomial and sigmoid approaches over the traditional mono-exponential function. Appropriately fitting the measured velocity data will have a significant impact on the FV metrics developed from the model fit.

Across all velocity-modelling approaches, there was agreement for $V_{max}$ and $V_{0}$ measures, indicating that all techniques equally evaluated the maximum and theoretical maximum velocity across all trials. The other four FV output metrics (${FV}_{slope},\;P_{max},\;F_{0},\;DRF$) all showed significant differences between velocity-fitting models. However, it is notable that the ${FV}_{slope},\;P_{max}$, and DRF measures are all either entirely or partially dependent on the determination of $F_{0\;rel}$ within the model. It was observed that all previously explored fitting techniques (both mono- and bi-exponential) consistently produced significantly higher $F_{0}$ values when compared to sigmoid and polynomial models. This result suggests that $F_{0}$ may be overestimated by exponential models, which in turn alters the dependent FV variables. Systematic modelling error, seen during the onset of the sprint for all three exponential functions where the maximum acceleration occurs, may be the root cause of this overestimation. For example, Furusawa’s mono-exponential model displayed larger error at the initial onset of the sprint, while the bi-exponential function had substantial error approaching the athlete’s maximum velocity. This may be due to underlying assumptions unique to each model, such as an initial velocity of zero or maximum acceleration at the onset of the sprint. These differences in modelled performance may have caused significant differences in FV measures, indicating that errors may exist when specific models are adapted to diverse athlete populations (i.e., skill level, sport background, and previous training), testing environments (i.e., indoor/outdoor, weather, sprinting surface, and footwear) and sprint protocols [12,13,17,24].

All of the velocity models explored in this study display a similar ability to detect changes in FV variables when subject to varying levels of sprint performance. Each modelling technique successfully displayed significant differences between the top and bottom 25% athlete groupings across all FV measures, indicating that they can all be used to detect change between performance levels. However, it is important to note that some metrics, such as $F_{0}$, show relatively high variability depending on the velocity model used. Further, each model demonstrated similar reactivity to subtle changes in sprint performance, with each technique detecting significant differences at the same percentage change step in trial velocity across all FV outputs, as shown in Table 2. These results indicate that all modelling methods react proportionally to similar changes in sprint performance. As a result, researchers and practitioners can reliably detect relative changes in performance with any of the five modelling techniques outlined in this paper. While exponential models have been substantiated in previous research, these findings support further inquiry into the use of the other fitting techniques, specifically the sigmoid and polynomial functions, for sprint-velocity modelling [6,17,27].

One point of differentiation between the sigmoid and polynomial models is the physiological and mechanical interpretation of model variables. Sigmoid functions have been previously used to model physiological phenomena and mechanical events, such as resistive force modelling [19,20]. Within the context of the linear sprint, sigmoid variables represent mechanical phenomena during the effort. Specifically, the variables L and b present in the sigmoid model, shown in Equation (7), represent the sprint’s maximum modelled velocity using Equation (8), while no such relationship exists for the polynomial function.

(8) $$V_{max}=L-b$$

Further, the variable $t_{0}$ represents a time delay, or time correction, which is shown in other velocity-modelling techniques such as the mono-exponential time-delay model discussed in this paper [12]. Finally, the sigmoid parameter k is an exponential multiple that is related to a participant’s acceleration throughout the sprint. Similar variables are present in all previously explored modelling techniques, either in the similar form of k or *t*. While polynomial fitting techniques have been used to model velocity previously [6,27], these physiological and mechanical underpinnings are not present and, therefore, may bring into question the biomechanical and physiological rationale behind their use and, consequently, the efficacy of the derived FV measures.

Based on the current investigation, we recommend the use of the sigmoid model for linear sprint-velocity fitting of team sport athletes. The flexibility of this model, in conjunction with its ability (but not requirement) to replicate the mono-exponential relationship in the sprint indicates its now-proven ability to better fit sprint-velocity data. Based on our findings, we recommend its general use for sprint testing. However, the current project was not without limitations. Firstly, our methodology used a fixed starting procedure and running surface for all of the sprint data collection. Future research is needed to explore the various kinematic differences between sprint starting techniques across running surfaces and starting techniques (i.e., blocks, three-point start, etc.) in order to adequately determine if our results can be generalized. Further, the population in this study, while including a wide variety of spiriting abilities, failed to include an elite sprinting population. Exploration of various sprint modelling techniques with an elite sprinting population would once again help support the generalizability of our findings.

## 5. Conclusions

The results of this study indicate that the sigmoid and polynomial velocity-fitting models display similar reliability and significantly better goodness-of-fit measures when compared to previously explored velocity-modelling techniques. Further, these modelling methods produced significantly different ${FV}_{slope},\;P_{max},\;DRF$, and $F_{0}$ FV outputs, suggesting systematic biases may be present in some previously explored FV velocity models. This may be due to underlying mathematical assumptions and limitations associated with the mono-exponential model. Specifically, during the onset of the sprint, this fitting error may result in an overestimation of maximum acceleration, which is a direct derivative of the velocity model on which force and FV measures are determined. Based on goodness-of-fit and model characteristic considerations, the sigmoid model provides the best velocity model for the 40 m sprint trials used in this study. Further exploration into the robustness of fit across other sprint applications is suggested to determine optimal fitting techniques for various sprint modalities and collection protocols.

## Figures and Tables

**Table 1:** Comparisons of means and standard error for six FV profiles and two goodness-of-fit measures across all five modelling techniques. Significant differences ( *p* < 0.05) between models are indicated using unique superscript, where Furusawa = F, Morin = M, Volkov = V, polynomial = P, sigmoid = S.

	Velocity Model				
Metric	Mono-Exponential	Mono-Exponential TD	Bi-Exponential	Polynomial	Sigmoid
P_max_ (W/kg)	12.231 ± 2.103	12.219 ± 2.105	11.515 ± 2.026	11.910 ± 2.129	12.222 ± 2.204
F_0_ (N/kg)	6.071 ± 0.635 ^V,P,S^	6.061 ± 0.637 ^V,P,S^	5.224 ± 0.542 ^F,M^	5.303 ± 0.562 ^F,M^	5.370 ± 0.667 ^F,M^
V_0_ (m/s)	8.062 ± 0.676	8.063 ± 0.676	8.199 ± 0.724	8.324 ± 0.718	8.262 ± 0.747
V_max_ (m/s)	7.651 ± 0.616	7.638 ± 0.617	7.559 ± 0.608	7.709 ± 0.629	7.594 ± 0.624
FV_Slope_	−0.754 ± 0.057 ^V,P,S^	−0.752 ± 0.057 ^V,P,S^	−0.638 ± 0.045 ^F,M^	−0.637 ± 0.042 ^F,M^	−0.652 ± 0.075 ^F,M^
DRF	−6.817 ± 0.462 ^V,P,S^	−6.841 ± 0.480 ^V,P,S^	−6.036 ± 0.412 ^F,M^	−5.975 ± 0.366 ^F,M^	−6.105 ± 0.627 ^F,M^
RMSE	0.191 ± 0.055 ^P,S^	0.191 ± 0.055 ^P,S^	0.185 ± 0.047 ^P,S^	0.147 ± 0.040 ^F,M,V^	0.139 ± 0.041 ^F,M,V^
R^2^	2.713 ± 0.266 ^P,S^	2.713 ± 0.266 ^P,S^	2.723 ± 0.262 ^P,S^	2.950 ± 0.272 ^F,M,V^	3.006 ± 0.287 ^F,M,V^

**Table 2:** Percentage differences in velocity data where significant differences occur for FV profile measures. Mean and standard error for modified and unmodified values also reported.

Model	P_Max_ (W/kg)			F_0_ (N/kg)			V_0_ (m/s)			V_Max_ (m/s)		
	%	Unmodified	Modified	%	Unmodified	Modified	%	Unmodified	Modified	%	Unmodified	Modified
Mono-exponential	3	12.48 ± 0.220	13.50 ± 0.238	4	6.13 ± 0.066	6.37 ± 0.068	3	8.15 ± 0.070	8.48 ± 0.073	3	7.73 ± 0.063	8.03 ± 0.066
Mono-exponential TD	3	12.47 ± 0.220	13.49 ± 0.238	4	6.12 ± 0.066	6.36 ± 0.069	3	8.15 ± 0.070	8.48 ± 0.073	3	7.71 ± 0.064	8.02 ± 0.067
Bi-exponential	3	11.75 ± 0.212	12.72 ± 0.230	4	5.28 ± 0.056	5.48 ± 0.058	3	8.28 ± 0.075	8.63 ± 0.078	3	7.63 ± 0.063	7.94 ± 0.066
Polynomial	3	12.15 ± 0.223	13.15 ± 0.241	4	5.36 ± 0.058	5.57 ± 0.061	3	8.41 ± 0.075	8.76 ± 0.078	3	7.79 ± 0.065	8.09 ± 0.068
Sigmoid	3	12.25 ± 0.230	13.49 ± 0.249	4	5.42 ± 0.069	5.64 ± 0.072	3	8.35 ± 0.077	8.69 ± 0.081	3	7.67 ± 0.065	7.97 ± 0.067

## Data Availability

Data used in this study were shared by University of Victoria Vikes and are not available for further sharing.
